# Parathyroid hormone levels and sarcopenia in dialysis patients: absence of association or methodological limitations?

**DOI:** 10.1590/2175-8239-JBN-2026-E013en

**Published:** 2026-08-07

**Authors:** Amanda Carina Coelho de Morais, Cássia Gomes da Silveira Santos, Fellype Carvalho Barreto

**Affiliations:** 1 Universidade Estadual de Maringá, Hospital Universitário Regional de Maringá, Maringá, PR, Brazil.; 2 Universidade Federal do Paraná, Complexo Hospital de Clínicas, Serviço de Nefrologia, Curitiba, PR, Brazil.; 3 Universidade Federal do Paraná, Programa de Pós-Graduação em Medicina Interna e Ciências da Saúde, Departamento de Clínica Médica, Curitiba, PR, Brazil.

Sarcopenia is a progressive and gen­eralized skeletal muscle disease associated with an increased likelihood of adverse outcomes, including falls, bone fractures, physical disability, and mortality^1^. Muscle mass loss is a prevalent complication of chronic kidney disease (CKD), particularly in stage 5D^2^. The relationship between CKD-mineral and bone disorder (CKD-MBD) and muscle impairment has attracted growing interest in recent years. In the current issue of the BJN, Leal et al. published the article entitled “Relationship Between Parathyroid Hormone Levels, Sarcopenia Status, and Nutritional Status in Hemodialysis Patients”, which addresses the potential interaction among secondary hyperparathyroidism (SHPT), nutritional status, and sarcopenia in hemodialysis patients, a clinically relevant yet still underexplored issue^3^.

Previous studies have reported that SHPT may contribute to sarcopenia and interfere with nutritional status. An experimental study in mice subjected to 5/6 nephrectomy demonstrated that PTH may play an important role in adipose tissue loss, increased energy expenditure, and muscle wasting^4^. An observational study including 42,319 patients undergoing chronic hemodialysis, derived from the DOPPS (Dialysis Outcomes and Practice Patterns Study), phases 2 to 6 (2002–2018), identified an inverse association between baseline PTH levels and 12-month weight loss, independent of hospitalization. This association was more pronounced among patients with higher PTH levels and those with preserved appetite^5^. Additionally, the authors demonstrated that the effect of the association between PTH ≥ 600 pg/mL and mortality could potentially be mediated by weight loss^5,6^. A retrospective study evaluating 410 patients on chronic hemodialysis, stratified according to PTH levels, observed that patients with severe SHPT (PTH ≥ 1,500 pg/mL) exhibited reduced muscle mass and poorer nutritional status, suggesting a direct relationship between PTH levels and the possible worsening of sarcopenia in dialysis patients^7^.

Leal et al. deserve recognition for investigating this issue in a relatively robust sample of hemodialysis patients, using current sarcopenia criteria established by the European Working Group on Sarcopenia in Older People^3^. The combined assessment of muscle strength, muscle mass, gait speed, and muscle quality index represents a methodological strength of the study. Another merit is the attempt to control for confounding factors through matching for age, sex, and the presence of *diabetes mellitus*. In studies addressing sarcopenia in CKD, this is particularly important given the impact of these factors on body composition and muscle function. Nevertheless, despite the clinical relevance of the study, some methodological aspects limit the interpretation of the findings and should be carefully considered.

One of the main critical issues relates to the cross-sectional design of the study, which precludes the establishment of causal relationships. A single-point assessment of PTH levels is unlikely to adequately reflect the cumulative disease burden of SHPT. Patients classified within the PTH ≥ 600 pg/mL group may have heterogeneous clinical histories, including either recent elevations or persistently elevated levels over many years. Furthermore, sarcopenia is a dynamic, progressive, and multifactorial condition, and the muscular consequences associated with SHPT likely depend on both the duration and intensity of exposure to elevated PTH levels. Moreover, considering that severe SHPT (PTH ≥ 1,500 pg/mL) may cause more severe and measurable muscular clinical consequences^7^, grouping all patients with PTH ≥ 600 pg/mL into a single category may have diluted the potentially more deleterious effects of higher PTH levels. Important determinants of sarcopenia in CKD patients, such as systemic inflammation, physical activity level, dialysis adequacy, protein-energy intake, and the use of active vitamin D or calcimimetics, were not evaluated. The absence of these variables limits the ability to perform adequate multivariable adjustment and hinders more robust conclusions, as acknowledged by the authors among the study limitations.

Regarding the method used to assess muscle mass, bioelectrical impedance analysis, although practical and widely available, has important limitations in dialysis patients. Even when measurements are performed after a hemodialysis session, residual alterations in volume status may significantly affect the assessment of lean body mass^8^. Furthermore, a national clinical study involving 49 patients with CKD suggested that bioelectrical impedance findings should be interpreted with caution in patients with SHPT, as elevated PTH and alkaline phosphatase levels may lead to greater bone loss and, consequently, greater discrepancies compared with dual-energy X-ray absorptiometry, which would provide greater phenotypic accuracy in this population^9^.

The study highlights an aspect frequently overlooked in clinical practice: the high prevalence of muscle impairment among CKD patients. Despite the relatively young mean age of the cohort (49 years), 18.5% of patients were sarcopenic. Low muscle quality was present in approximately two-thirds of the sample. These findings reinforce the need to incorporate nutritional and physical functional assessments into routine care within dialysis centers. Additionally, the discussion regarding publication bias acknowledges that studies reporting negative findings are less likely to be published. This position demonstrates the authors’ critical awareness of the scientific value of divergent results, which contribute to refining clinical hypotheses.

This study adds original data to the field by exploring the association between PTH levels and sarcopenia in Brazilian patients undergoing hemodialysis using contemporary diagnostic criteria. Although methodological limitations preclude definitive conclusions, the study reinforces the complexity of sarcopenia in CKD and highlights the need for longitudinal studies incorporating serial PTH assessments, stratification of SHPT severity, and the inclusion of inflammatory and functional biomarkers. More than providing definitive answers, the study contributes to placing CKD-MBD at the center of the discussion on sarcopenia in CKD.

## Data Availability

No new data were generated during the development of this study.
