# Supplementary material for: Parathyroid hormone levels and sarcopenia in dialysis patients: absence of association or methodological limitations?
Source: J Bras Nefrol. 2026 Aug 7;48(3):e2026E013. doi: 10.1590/2175-8239-JBN-2026-E013en (PMC13456425; doi:10.1590/2175-8239-JBN-2026-E013en)
Supplement: Translation

# Translation: Níveis de paratormônio e sarcopenia em pacientes dialíticos: ausência de associação ou limitações metodológicas?

**DOI:** 10.1590/2175-8239-JBN-2026-E013pt

**Authors:** Amanda Carina Coelho de Morais, Cássia Gomes da Silveira Santos, Fellype Carvalho Barreto

**Affiliations:** 1 Universidade Estadual de Maringá, Hospital Universitário Regional de Maringá, Maringá, PR, Brasil.; 2 Universidade Federal do Paraná, Complexo Hospital de Clínicas, Serviço de Nefrologia, Curitiba, PR, Brasil.; 3 Universidade Federal do Paraná, Programa de Pós-Graduação em Medicina Interna e Ciências da Saúde, Departamento de Clínica Médica, Curitiba, PR, Brasil.

A sarcopenia é uma doença muscular esquelética progressiva e generalizada associada a uma maior probabilidade de desfechos adversos, incluindo quedas, fraturas, incapacidade física e mortalidade^1^. A perda de massa muscular é uma complicação prevalente na doença renal crônica (DRC), especialmente no estágio 5D^2^. A relação entre o distúrbio mineral e ósseo da DRC (DMO-DRC) e o comprometimento muscular tem despertado crescente interesse nos últimos anos. Na edição atual do BJN, foi publicado o artigo intitulado “Relação entre níveis de paratormônio e *status* de sarcopenia e estado nutricional em pacientes em hemodiálise”, de Leal et al., que aborda a possível interação entre hiperparatireoidismo secundário (HPTS), estado nutricional e sarcopenia em pacientes com DRC em hemodiálise, uma questão clínica relevante e ainda pouco explorada^3^.

Estudos prévios reportaram que o HPTS pode contribuir para a sarcopenia e interferir no estado nutricional. Um estudo experimental em camundongos submetidos à nefrectomia 5/6 revelou que o PTH pode ter um importante papel na perda de tecido adiposo, no aumento do gasto energético e na perda muscular^4^. Um estudo observacional que incluiu 42.319 pacientes em hemodiálise crônica, provenientes do estudo DOPPS (*Dialysis Outcomes and Practice Patterns Study*), fases 2 a 6 (2002–2018), identificou uma associação inversa entre o nível de PTH, no início do estudo, e a perda ponderal em 12 meses, independentemente de hospitalização, a qual foi mais pronunciada nas faixas de níveis mais elevados de PTH e entre aqueles com apetite preservado^5^. Adicionalmente, demonstraram que o efeito da associação entre PTH ≥ 600 pg/mL e mortalidade poderia ser potencialmente mediado pela perda de peso^5,6^. Um estudo retrospectivo, que avaliou 410 pacientes em HD crônica estratificados conforme os níveis de PTH, observou que indivíduos com HPTS grave (PTH ≥ 1.500 pg/mL) apresentavam redução da massa muscular e pior estado nutricional, sugerindo uma relação direta entre os níveis de PTH e o possível agravamento da sarcopenia em pacientes dialíticos^7^.

Leal et al. merecem reconhecimento por investigarem essa questão em uma amostra relativamente robusta de pacientes em HD, utilizando critérios atuais de sarcopenia definidos pelo *European Working Group on Sarcopenia in Older People* ^3^. A avaliação combinada de força muscular, massa muscular, velocidade de marcha e índice de qualidade muscular representa um ponto forte metodológico do estudo. Outro mérito é a tentativa de controle de fatores de confusão, por meio do pareamento por idade, sexo e presença de *diabetes mellitus*. Em estudos sobre sarcopenia na DRC, isso é particularmente importante, considerando o impacto dessas variáveis sobre a composição corporal e a funcionalidade muscular. Entretanto, apesar da relevância clínica do estudo, alguns aspectos metodológicos limitam a interpretação dos resultados e devem ser considerados cuidadosamente.

O principal ponto crítico refere-se ao desenho transversal do estudo, que impede estabelecer relações causais, inerentes à metodologia. Dessa forma, uma avaliação pontual do PTH provavelmente não reflete adequadamente a carga cumulativa da doença associada ao HPTS. Pacientes classificados no grupo de PTH ≥ 600 pg/mL podem ter histórias clínicas heterogêneas, incluindo níveis elevados recentes ou persistentes por muitos anos. Além disso, a sarcopenia é uma condição dinâmica, progressiva e multifatorial, cuja repercussão muscular relacionada ao HPTS possivelmente depende da duração e intensidade da exposição ao PTH elevado. Considerando que casos de HPTS grave (PTH ≥ 1.500 pg/mL) causam repercussões clínicas musculares mais graves e mensuráveis^7^, o agrupamento de todos os pacientes com PTH ≥ 600 pg/mL em uma única categoria pode ter diluído a observação dos efeitos deletérios do PTH mais elevado. Fatores relacionados à sarcopenia em pacientes com DRC, como inflamação sistêmica, nível de atividade física, adequação dialítica, ingestão proteico-calórica, além do uso de vitamina D ativa ou de calcimiméticos, não foram avaliados. A ausência dessas variáveis limita a capacidade de ajuste multivariado e dificulta conclusões mais robustas, conforme os próprios autores reconhecem entre as limitações do estudo.

Em relação ao método utilizado para avaliação da massa muscular, a bioimpedância elétrica, embora prática e amplamente disponível, apresenta limitações relevantes em pacientes dialíticos. Ainda que as medidas tenham sido realizadas após a sessão de hemodiálise, alterações no estado volêmico residual podem impactar significativamente a avaliação da massa magra^8^. Além disso, um estudo clínico nacional com 49 pacientes com DRC sugere que a bioimpedância deve ser interpretada com cautela em pacientes com HPTS, uma vez que níveis elevados de PTH e fosfatase alcalina podem levar à maior perda óssea e, consequentemente, a uma maior discrepância em comparação com a densitometria óssea por absorciometria de raios X de dupla energia, que forneceria maior precisão fenotípica nesse grupo de pacientes^9^.

O estudo também chama a atenção para um aspecto frequentemente negligenciado na prática clínica: a elevada prevalência de comprometimento muscular nos pacientes com DRC. Apesar da idade média relativamente jovem (49 anos), 18,5% dos pacientes apresentavam sarcopenia. A baixa qualidade muscular estava presente em aproximadamente dois terços da amostra. Esses dados reforçam a necessidade de incorporação da avaliação nutricional e da avaliação física funcional nas unidades de diálise. Adicionalmente, a discussão sobre o viés de publicação reconhece que estudos com resultados negativos têm menor probabilidade de publicação. Esse posicionamento demonstra a consciência crítica dos autores quanto ao valor científico de resultados divergentes, que contribuem para refinar hipóteses clínicas.

O estudo acrescenta dados originais à literatura, explorando a associação entre PTH e sarcopenia em pacientes brasileiros em hemodiálise, utilizando critérios diagnósticos atuais. Embora limitações metodológicas impeçam conclusões definitivas, o estudo reforça a complexidade da sarcopenia na DRC e destaca a necessidade de investigações longitudinais, com avaliação seriada do PTH, estratificação da gravidade do HPTS e incorporação de biomarcadores inflamatórios e funcionais. Mais do que fornecer uma resposta definitiva, o estudo contribui para colocar o DMO-DRC no centro da discussão sobre sarcopenia na DRC.

## Data Availability

Não foram gerados novos dados durante o desenvolvimento deste estudo.
